# Peste des Petits Ruminants Virus Surveillance in Domestic Small Ruminants, Mozambique (2015 and 2017)

**DOI:** 10.3389/fvets.2019.00370

**Published:** 2019-11-08

**Authors:** Lourenço Mapaco, Iolanda Monjane, José Fafetine, Dercília Arone, Alexandre Caron, Abel Chilundo, Carlos Quembo, Maria Do Carmo Carrilho, Virginia Nhabomba, Siamak Zohari, Sara Achá

**Affiliations:** ^1^Agricultural Research Institute of Mozambique, Central Veterinary Laboratory, Maputo, Mozambique; ^2^ASTRE, CIRAD, INRA, University of Montpellier, Montpellier, France; ^3^Veterinary Faculty, Eduardo Mondlane University, Maputo, Mozambique; ^4^Ministry of Agriculture and Food Security, Veterinary National Directorate, Maputo, Mozambique; ^5^National Veterinary Institute, Department of Microbiology, Uppsala, Sweden

**Keywords:** PPR virus, small ruminants, surveillance, Mozambique, antibody, RT-PCR

## Abstract

Peste des Petits Ruminants (PPR), a transboundary animal disease affecting mainly goats and sheep is caused by a morbillivirus and threatens food security and livelihoods as morbidity and mortality rates can reach 90%. There are no records of PPR in Mozambique, but the disease situation in Tanzania and the ability of PPR virus to rapidly spread across countries constitute a high risk for about 4.7 million goats and sheep in Mozambique. A total of 4,995 goats and sheep were sampled in several provinces during 2015 and 2017 to assess the status of PPR virus (PPRV) in Mozambique and to contribute to surveillance along the border with Tanzania. The sera were screened for anti-PPRV antibodies using a commercial PPR competition ELISA (c-ELISA) and the haemagglutinin based PPR blocking ELISA (HPPR-bELISA). The swabs were tested using one-step RT-PCR for detection of PPRV RNA. The overall percentage of animals with anti-PPRV antibodies by c-ELISA, was 0.46% [0.30–0.70]. However, all the sera positive on c-ELISA were confirmed to be negative by the HPPR-bELISA. Considering that all the swabs were negative for detection of PPRV, no clinical cases were observed during passive surveillance and active sampling, and no symptoms were reported, these results suggest that PPRV is not present in Mozambique.

## Introduction

Peste des Petits Ruminants (PPR), a transboundary animal disease affecting mainly goats and sheep, is a highly contagious small ruminant's disease with significant economic impacts due to the high morbidity and mortality rates ranging from 10–90% and 50–90%, respectively, in naive populations. The disease is caused by a morbillivirus, a single-stranded RNA virus of the family *Paramyxoviridae*, a virus related to the now eradicated Rinderpest virus (1). Once closely associated with the latter in African ruminant populations, triggering cross-immunity and cross-reaction between both viruses, PPR now ranges freely on the African continent and has been spreading since the late 1990s, early 2000s.

The epidemiology of PPR in domestic animals is globally understood (2, 3). In endemic areas, morbidity and mortality can be much lower, blurring the epidemiological picture. The classical clinical expression of the infection includes watery nasal and lachrymal discharges, fever and at later stage diarrhea and coughing. Differential diagnosis can be difficult in African contexts where multiple infections are co-occurring, sometimes simultaneously, in small ruminant populations [e.g., bluetongue, foot and mouth disease (FMD), contagious caprine pleuropneumonia, brucellosis, rift valley fever, or Q fever] (4, 5).

The history of the geographical spread of PPR in Africa is not entirely understood. Endemic for a long time in Western and the Sahelian part of Central Africa, the disease spread to East Africa at the beginning of the twenty-first century, emerging first in Uganda, probably spreading from the then Sudan to subsequently reach Kenya and Tanzania (2). Four lineages (I-IV) are present in Africa with lineage IV being a new invasive strain from the Middle East and Asia, replacing other strains. The recent spread and mixing of lineages, notably in Tanzania could confuse the disease geography and clinical patterns. Tanzania is now endemic for PPR, potentially hosting at least three of the four existing lineages and with a widespread presence of the infection and disease across its territory (6).

In southern Africa, the disease has spread in new areas in recent years. Tanzania represents a significant potential source of PPR viruses for the rest of the region. Tanzania has a large small ruminant population and is engaged in trade with its neighbors, exporting formally or informally large numbers of small ruminants. The disease has already spread from Tanzania to Democratic Republic of Congo (DRC) and Comoros (7), but so far, it has not been reported and confirmed in Malawi, Mozambique, and Zambia. The borders between Tanzania and these last two countries therefore constitute an important entry gate for PPR into the rest of southern Africa, and surveillance and control need to be implemented in order to prevent the disease from spreading further southward, where it could infect not only the countries with a common border (Malawi, Mozambique, and Zambia), but also Botswana, Lesotho, Namibia, South Africa, Swaziland, and Zimbabwe. As demonstrated for other transboundary animal diseases such as FMD, once the virus enters a country such as Mozambique or Zambia, it can easily spread within the region (8). This is due firstly to the extensive informal trade in small ruminants occurring amongst southern African countries and secondly due to the promotion of wildlife population connectivity in the region through the creation of Transfrontier Conservation Areas for the last 20 years. African ungulates, particularly antelopes, are susceptible to the infection but no disease has been reported so far in those species (9), while a recent outbreak in Central Asian ungulate species raises the concern of the impact of PPR on threatened species (10). The role of wildlife in the epidemiology of PPR is not yet fully clarified, and it cannot be excluded that wildlife could spread the disease across borders (11).

In Africa, the disease represents a threat to the livelihoods of some of the most vulnerable and poor communities. Small-scale farmers, notably women largely involved in the small ruminant economy, rely heavily on small ruminants for income, assets, nutrition and health as well as soil management (12). For these reasons, PPR has been identified as a target for control by OIE and FAO with an objective to eradicate the disease worldwide by 2030. However, for the implementation of better PPR control strategies, it will be important to improve our knowledge in epidemiology, genetics, pathogenicity, and virulence characteristics of the virus.

Mozambique shares borders with Tanzania, with endemic PPR, Malawi, South Africa, Zambia, and Zimbabwe with no record of clinical PPR. However, like Mozambique, Zambia, and Malawi are classified as high-risk countries for PPR introduction given their shared borders with Tanzania, DRC, and Angola, all infected countries. Mozambique has a small ruminant population of ~4.7 million heads, most of them produced in the Central and southern regions. Important wild ungulate populations inhabit large national parks and reserves in all provinces of Mozambique and these populations could play a role in the epidemiology of the disease. The interface between these wildlife populations and livestock has not been characterized and the risk of PPR introduction or spread through the wildlife population is unknown. The country has engaged in the Progressive Step-wise Approach for the prevention and control of PPR (13). To address these needs, an intensive clinical and sero-epidemiological survey of the disease was carried out in Mozambique. In this context, this study reports a clinical, serological, and virological survey in 5 provinces of Mozambique, where the risk of disease transmission could be present, in order to provide information about the PPR status of the country.

## Materials and Methods

### Study Area

**Figure 1:**
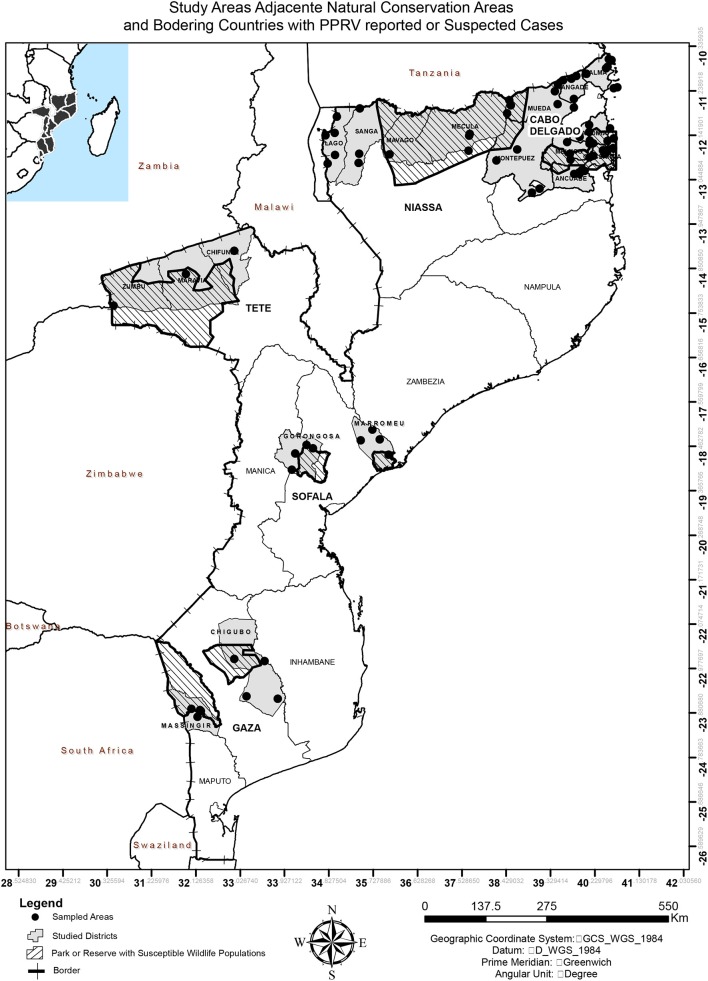
Map of Mozambique indicating the study areas, adjacent natural conservation areas, and bordering countries with PPRV reported or suspected cases.

The study was conducted in 5 of the 10 Provinces of Mozambique, from the three geographic regions (Figure 1). Provinces were selected on the basis of two criteria: sharing a border with Malawi, Tanzania, or Zambia (infected or high-risk country) and hosting national park or reserve with susceptible wildlife populations. In the north, Cabo Delgado and Niassa were selected because they share borders with Tanzania and due to the presence of susceptible wildlife populations in Niassa National Reserve, which covers some districts from both provinces, and Quirimbas National Park in Cabo Delgado. In the Centre, Tete province was part of the study due to PPRV suspected cases in 2015 in Zambia (only positive serology detected, no disease ever reported), while Gorongosa National Park and Marromeu National Reserve were the criteria for Sofala's inclusion. In the South, Gaza was selected due to the existence of Limpopo National Park and Banhine National Park. The study was performed with permission of the National Veterinary Directorate, Ministry of Agriculture and Food Security of Mozambique.

### Sampling

A longitudinal study consisting of two cross sectional surveys was carried out across 2015 and 2017. The districts then villages were selected taking into account the density of small ruminants provided by local key informants (mainly district staff from the Department of Veterinary Services) and, then, the accessibility of the site, as some villages are not reachable by car. Finally, inside each village, herds were selected based on the willingness of owners to participate in the study. Therefore, due to these constraints the sampling methodology was a convenient sampling. For herds with <10 animals all were sampled while in case of herds with more than 10 animals, at least 10% of animals were included. Breeds were not considered/identified in this study, just species because the so called small-scale farmers normally keep the indigenous “breeds” that comprise cross- and non-characterized breeds.

The blood samples were collected into plain vacutainer tubes and kept at room temperature for clotting to obtain sera. Ten percent of nasal swabs were intentionally taken from the same population and preserved in Phosphate Buffer Saline (PBS) containing antibiotics (kanamycin, streptomycin, and tetracycline).

### Laboratory Testing

Two serological tests were performed to detect PPRV antibodies. First the sera were screened for anti-PPRV antibodies using a commercially available competitive ELISA (c-ELISA) kit (ID-Vet ID Screen® PPR Competition) for the detection of anti-PPRV nucleoprotein antibodies in sheep and goat serum or plasma (14), following the manufacturer's instructions. While analyzing the results we received notification from the kit manufacturer about the specificity shift of the batch that was at that time available on the market (B78) and was being used in this study. Based on the recommendations of the manufacturer and the European Reference Laboratory for PPRV, EURL-PPRV at the CIRAD, and an in-house re-evaluation of the specificity of the batch B78 using sera tested simultaneously with both c-ELISA batch B78 and D52, we interpreted the results with the batch B78 with the following modification; cut-off values: ≤ 30%: positive; >30% and <35%: doubtful and ≥35%: negative.

All the c-ELISA positive sera were then tested by HPPR-bELISA kit from the Pan African Veterinary Vaccine Centre of the African Union (15) for further confirmation following the manufacturer's instructions.

The swabs from all animals positive on c-ELISA were tested for the presence of PPRV nucleic acid using one-step RT-PCR. The swabs from 2015 were tested using a conventional reverse transcription polymerase chain reaction (RT-PCR) described in the OIE Manual (16), while swabs from 2017 were screened using a real-time reverse transcription polymerase chain reaction, targeting the PPRV N gene (17).

### Statiscal Analysis

All data were entered in MS Excel (Microsoft Corporation) spreadsheet and exported to SPSS version 12.1® (Stata IC 12.1 for Windows), software for analysis. Descriptive statistics were based on frequencies and percentages for qualitative variables and means and confidence intervals for quantitative variables. Prevalence data were calculated using either Fisher's exact test or the χ^2^-test.

Data generated were entered in Microsoft Excel and analyzed using descriptive statistics. The odds ratio (OR) was calculated to assess the association between being positive for PPR and reusing serological data. The OR assesses the association of being seropositive for PPR where *p* < 0.05 was considered as significant.

## Results

A total of 4,995 blood samples were collected from 4,315 goats and 680 sheep (Table 1) of different ages and breeds (mainly indigenous), between June and September 2015 and May and November 2017. The sera were analyzed for the presence of anti-PPRV antibodies using c-ELISA, and the overall percentage of positive sera was 0.46% [0.30–0.70]. Positive sera were found across all sampled provinces excluding Tete (Table 1). The positive sera on c-ELISA re-tested by HPPR-bELISA were all negative. The PPRV RNA was not detected in swabs submitted to molecular testing. During the sampling, the animals were inspected and no clinical signs resembling PPR infection were seen or reported.

**Table 1:** c-ELISA results (prevalence).

**Province**	**District**	**Year**	***N***	**Nr. of + (%)**	**[95% CI]**
Gaza	Massingir	2017	392	3 (0.77)	[0.20–2.41]
	Chigubo	2017	311	1 (0.32)	[0.02–2.06]
	Total		703	4 (0.57)	[0.18–1.55]
Tete	Chifunde	2015	151	0	–
	Marávia	2015	114	0	–
	Zumbo	2015	82	0	–
	Total		347	0	–
Sofala	Gorongosa	2017	246	1 (0.41)	[0.02–2.60]
	Marromeu	2017	400	3 (0.75)	[0.19–2.36]
	Total		646	4 (0.62)	[0.20–1.69]
Cabo Delgado	Quissanga	2017	278	1 (0.36)	[0.02, 2.30]
	Macomia	2017	338	1 (0.30)	[0.02–1.90]
	Palma	2015	131	0	–
		2017	248	2 (0.81)	[0.14–3.20]
	Mueda	2015	142	1 (0.70)	[0.14–3.20]
		2017	235	5 (2.13)	[0.79–5.17]
	Montepuez	2017	147	2 (1.36)	[0.24–5.33]
	Ancuabe	2017	293	0	–
	Meluco	2017	163	0	–
	Nangade	2015	130	0	–
	Total	2015	403	1 (0.25)	[0.01–1.60]
		2017	1,702	11 (0.65)	[0.34–1.19]
	Total		2105	12 (0.57)	[0.31–1.02]
Niassa	Mecula	2015	101	0	–
		2017	139	1 (0.72)	[0.04–4.54]
	Mavago	2015	132	0	–
		2017	231	1 (0.43)	[0.02–2.76]
	Sanga	2015	39	0	–
		2017	17	0	–
	Lago	2015	103	1 (0.97)	[0.05–6.07]
		2017	432	0	–
	Total	2015	375	1 (0.27)	[0.01–1.71]
		2017	819	2 (0.24)	[0.04–0.98]
	Total		1,194	3 (0.25)	[0.06–0.80]
Total		2015	1,125	2 (0.18)	[0.03–0.71]
		2017	3,870	21 (0.54)	[0.34–0.84]
Total			4,995	23 (0.46)	[0.30–0.70]

## Discussion

PPR is an epizootic disease of small ruminants causing high morbidity and mortality in affected animals, constituting a significant threat to livestock production, and represents a danger to food security in developing countries due to mortality rates that can reach 100% (18). PPR outbreaks have major socioeconomic implications for farmers and agricultural sectors, especially in countries where small ruminants play an integral role in sustainable agriculture and employment, thereby contributing to an increase in poverty in regions with dominant dependence on farming small ruminants.

Traditional livestock trade routes exist between all neighboring countries of Mozambique, although their frequency and intensity have not been measured. Mozambique shares its northern border with the United Republic of Tanzania, a country in which PPR is endemic. The risk of PPR introduction from known infected areas in Tanzania into Mozambique is considered to be high due to this transboundary trade and transport of small ruminants even if its extent is unknown. The Mozambican borders with Zambia and Malawi are considered at lower risk because no clinical disease has ever been reported in these 2 countries. However, the Tete region has a high density and trade of small ruminants and should be specifically targeted for surveillance. Other areas targeted by this study in Sofala and Gaza present a lower risk of PPR circulation because neighboring countries (i.e., South Africa, Swaziland, and Zimbabwe) are far from the nearest outbreaks (in Tanzania and DRC). The presence of large populations of wildlife in some protected areas in these provinces can be a risk factor for PPR circulation because the role of wildlife in PPR epidemiology is largely unknown. Wildlife populations are known to be exposed to the virus in East Africa but no clinical disease has ever been observed in wildlife in Africa.

While serological tests are designed to be sensitive and specific, false positive and false negative results do occur; therefore, it is strongly recommended to confirm any new positive finding by using alternative diagnostic methods.

Positive serum samples were found in four provinces out of five sampled and the global prevalence was 0.46 [0.30–0.70]. The differences between the provinces at high risk (Niassa and Cabo Delgado) and those of medium (Tete) and low risk (Sofala and Gaza) was not significant (*p* = 0.543).

The c-ELISA test we used has 99.4% of specificity (14), therefore, the 0.46% we detected in our study is within the expected level for non-infected population. Global seroprevalences of 57.6% in Uganda (19), 48.5% in Pakistan (20), 45.66% in sheep and 38.54% in goats in India (21) and of 45.4, 31.0, and 27.1% in 2009, 2012, and 2015, respectively, in Tanzania (5, 6) have been reported by using c-ELISA test. These studies showed a high seroprevalence because the samples tested were from animals exposed to the virus with or without clinical signs of the disease.

For better interpretation of our results, a confirmation by the HPPR-bELISA with 100% specificity compared to Virus Neutralization Test (15), was performed. All seropositive samples by c-ELISA came out negative with the HPPR-bELISA, invalidating the seroconversion detected by c-ELISA. Our data indicate an overall seronegativity of the sampled populations. In addition to this serosurveillance data, there has never been any reported outbreak of PPR in small ruminants in Mozambique. No official vaccination has been carried out against PPR in Mozambique. As small ruminant populations were expected to be naïve to PPR virus infection, PPRV incursion would result in high morbidity and mortality in the non-vaccinated naïve population of small ruminants in Mozambique.

Low PPR prevalence in non-infected countries can be possible for several reasons. Low level PPRV antibodies in this study detected by the c-ELISA may result from: (i) imported animals from infected countries that have been infected and survived the disease; (ii) imported animals from infected countries that have been vaccinated against PPR; (iii) False positives due to the specificity of the c-ELISA test. In the first two cases, seropositivity would be detected preferentially in areas bordering an infected area (i.e., Tanzania), which is not what has been observed here. However, the likelihood of finding positive animals seemed to be higher for low risk provinces than the high risk ones OR = 1.306 [0.552–3.088]. These findings are somehow contradictory taking into account that the major risk factor of PPRV introduction into Mozambique is the disease situation in Tanzania. Finally, given the size of the sampling, false positives are expected and an absence of seropositivity through c-ELISA screening would be suspicious.

PPRV is highly infectious, often spreading rapidly between groups of susceptible animals, causing disease with distinct clinical signs. Thus, a lack of reports of clinical signs, the absence of RT-PCR positive results and the absence of seroprevalence after double-testing of samples among examined animals indicate that the virus does not actively circulate in the studied populations in Mozambique. These data can enable Mozambique to move forward in its Progressive Control Pathway toward OIE PPR free status. However, this status should not hide the fact that Mozambique is a country at high-risk of contracting PPR due to its border with Tanzania. In addition, the presence of large susceptible wildlife populations sharing space with livestock in the periphery of protected areas is an additional risk factor that should be taken into account (9). In the future, targeted or opportunistic (e.g., for conservation translocation) sampling could be useful to assess the risk of wildlife introducing PPR across border or spreading it between provinces.

Mozambique, together with Malawi, Zambia, and Namibia, should strengthen its surveillance system in border areas. A risk-based approach taking into account small ruminant movements across the Tanzanian-Mozambican border should help in designing a reactive passive surveillance system.

## Data Availability Statement

The datasets generated for this study are available on request to the corresponding author.

## Ethics Statement

The animal study was reviewed and approved by Veterinary National Directorate, Mozambique. Written informed consent was obtained from the owners for the participation of their animals in this study.

## Author Contributions

SA, SZ, and ACa conceived and designed the study, organized protocol developments, contributed to the conception and interpretation of the data, and revised the manuscript. LM developed the sampling design, directed the collection of samples, carried out field sampling and laboratory investigations, interpreted the data, organized the dataset, and wrote the first draft of the manuscript. IM, VN, and DA carried out field sampling and laboratory investigations. ACh performed statistical analysis, contributed to the interpretation of the findings, and revised the manuscript. CQ, JF, and MC contributed to the interpretation of the findings and revised the manuscript.

### Conflict of Interest

The authors declare that the research was conducted in the absence of any commercial or financial relationships that could be construed as a potential conflict of interest.
